# Development of a new High Resolution Melting (HRM) assay for identification and differentiation of *Mycobacterium tuberculosis* complex samples

**DOI:** 10.1038/s41598-018-38243-6

**Published:** 2019-02-12

**Authors:** Patricia Landolt, Roger Stephan, Simone Scherrer

**Affiliations:** 1 Institute of Veterinary Bacteriology, Vetsuisse Faculty, University of Zurich, Winterthurerstrasse 270, 8057 Zurich, Switzerland; 2 Institute for Food Safety and Hygiene, Vetsuisse Faculty, University of Zurich, Winterthurerstrasse 272, 8057 Zurich, Switzerland

## Abstract

The rapid identification and differentiation of members of the *Mycobacterium tuberculosis* complex (MTBC) is essential to assess the potential zoonotic risk. Different available molecular methods are time consuming since they depend on cultivation of mycobacteria. High Resolution Melting (HRM) is a low cost, rapid and easy to perform single-tube method not limited to cultured samples. In this study, a HRM assay specifically targeting *gyrB* was developed to simultaneously identify and differentiate *Mycobacterium (M.) tuberculosis*, *M. microti* and *M. bovis/M. caprae*. To evaluate the performance of this assay, 38 MTBC isolates and 25 directly extracted clinical specimens were analysed. HRM results of all 38 (100%) examined isolates correlated with the results obtained with the commercially available GenoType MTBC test (Hain Lifescience). From the 25 clinical specimens tested, species identification by HRM showed concordant results with the previously used identification methods in 23 samples (92%). The assay demonstrated a good analytical sensitivity, specificity and reproducibility and can be used directly on clinical specimens.

## Introduction

*Mycobacterium tuberculosis* complex (MTBC) consists of the closely related species *Mycobacterium (M.) tuberculosis, M. bovis, M. bovis* Bacillus Calmette and Guérin (BCG), *M. caprae, M. africanum, M. microti, M. pinnipedii, M. canettii* and three further species (*M. orygis*, the dassie bacillus, *M. mungi*)^1^. *M. tuberculosis* and *M. africanum* are often described as host-specific to humans. According to the recent WHO report from 2017, tuberculosis is still the leading cause of human death from a single infectious agent with 6.3 million new cases and an estimated 1.7 million deaths in 2016^2^. Additionally, several cases of *M. tuberculosis* infections in animals have been reported^3–5^. Bovine tuberculosis (bTB) is an important zoonosis most commonly caused by *M. bovis* and less frequently by *M. caprae*^1^. New cases of bTB in 2013 in Switzerland^6^, resulting in the implementation of a national bTB surveillance program, highlighted the importance of routine species-level identification. This program consists of a systematic microbiological testing of suspicious lymph nodes found linked to meat inspection in slaughterhouses. Fast and reliable identification and differentiation between the species within this MTB complex is important to assess the potential zoonotic risk and is therefore a fundamental procedure for public health surveillance and food safety.

Identification of MTBC is based on different PCR based methods targeting 16S rRNA or IS*6110*^7,8^. In contrast, differentiation of *Mycobacterium* species within the MTBC is more laborious. Diverse molecular methods such as the GenoType MTBC test (Hain Lifescience, Nehren, Germany) or restriction fragment length polymorphism^9,10^ are used although having limitations in the reliance on bacterial cultures to produce a valid amount of bacterial DNA. Mycobacteria especially *M. microti* requires several months to obtain significant growth underlining a delayed time span to get valid results of MTBC species-level identification. Spoligotyping uses polymorphism on the direct repeat locus for differentiation and typing of MTBC^11^. A result is obtained in one or two days comprising several working steps such as PCR, membrane preparation, hybridisation, washing steps and detection by chemiluminescence. On the other hand, newer alternatives to conventional spoligotyping such as Luminex technology or Spoligotyping by MALDI-TOF MS requires advanced and expensive equipment^12^. Numerous single-tube multiplex RT-PCR assays have been previously proposed for MTBC species discrimination, mainly targeting several region of differences (RD)^13–17^. The complexity of a multiplex RT-PCR reaction can be a problem for intricate veterinary samples often dealing with animal tissues.

High resolution melting (HRM) assay is reported as a rapid and low cost assay to detect single-nucleotide polymorphism (SNP)^18^. The assay characterizes amplified PCR products according to their dissociation behaviour without requiring additional instrumentation. A fluorescently labelled dye binding to double stranded DNA is combined with amplicons resulting from the PCR reaction. When increasing the temperature, the double stranded DNA dissociates into single strands leading to a decrease in fluorescence intensity. The melting temperature depends on GC content, length and nucleotide sequence. It is an easy to perform and single-tube method leading to a result within approximately two hours. Moreover, HRM is not limited to cultured material but is able to detect DNA in clinical specimens directly extracted from tissue samples. Various HRM assays are already used successfully to identify and differentiate many bacteria species such as *Mycoplasma synoviae*^19^, *Chlamydiaceae* sp.^20^, *Staphylococcus aureus*^21^, *Brucella* sp.^22^ and *Leptospira* sp.^23^. Among *Mycobacteria* sp. HRM is a tool often reported to analyse drug resistance in *M. tuberculosis*^24–26^. Furthermore, the method is used to differentiate non-tuberculous mycobacteria (NTM) and to distinguish them from MTBC^27–29^. Wright *et al*. designed a HRM assay to detect the Region of Deletion 9 (RD9) with the aim of differentiating *M. tuberculosis* from other members of MTBC in fine-needle aspiration biopsies^30^. However, the drawback of this study lies in its poor sensitivity of only 51.9%. Other studies combined RD-targeted multiplex RT-PCR assays with HRM^14,17^. In this study, a novel HRM method based on SNPs located in *gyrB* was developed to simultaneously identify and differentiate members of MTBC to its species-level.

## Results

### HRM Analysis with Cultured Samples

**Figure 1:**
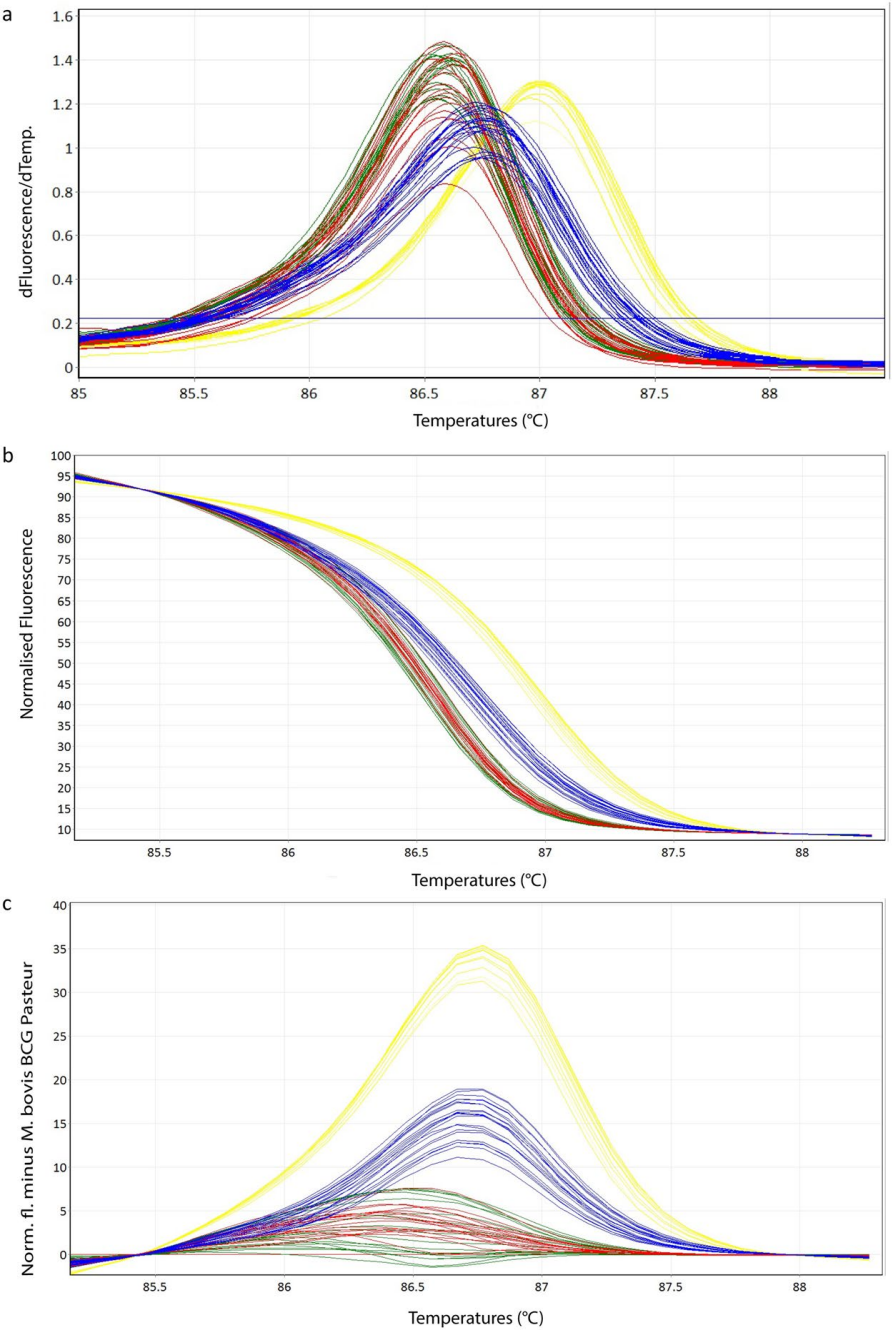
Representative high resolution melting graphs corresponding to one high resolution melting analysis of a subset of cultured samples (n = 22). Curves of tested samples previously identified as *M. tuberculosis* are shown in yellow, *M. microti* in blue, *M. bovis/M. bovis* BCG in red and *M. caprae* in green. (**a**) Melting curves; (**b**) Normalized plot; (**c**) Difference plot.

All cultured samples tested were amplified and resulted in a corresponding melting curve. The HRM species-specific melting temperatures T_m_ (Table 1, Supplementary Table S1) and the corresponding melting curves (Fig. 1a) of the sample-subset used for determination of the intra- and inter-assay reproducibility are shown. Since the T_m_ ranges are very close to each other, it is difficult to clearly distinguish between members of MTBC. On the other hand, the difference plot (Fig. 1c) as well as the normalized plot (Fig. 1b) allowed a clear species differentiation into the three groups of *M. tuberculosis*/*M. africanum, M. microti* and *M. bovis/M. caprae*. The intra-assay coefficiences of variation (CVs) and the inter-assay CVs showed values ranging between 0.01–0.02% and 0.02–0.03%, respectively (Table 1, Supplementary Table S1). Species identification results of all 38 (100%) tested cultured samples correlated with the GenoType MTBC test (Hain Lifescience) results.

**Table 1:** Melting temperatures (mean and standard deviation) of the intra- and inter-assay of a randomly chosen subset of cultured samples for different MTBC species with its corresponding coefficients of variation (CV) in % are listed.

	Run 1		Run 2		Run 3		Inter-Assay	
	CV%	Tm values	CV%	Tm values	CV%	Tm values	CV%	Tm values
*M. tuberculosis* H37Rv		86.93		86.98		86.95		
*M. bovis* BCG Pasteur ATCC 35734		86.60		86.58		86.58		
*M. microti* ATCC 19422		86.75		86.72		86.73		
*M. tuberculosis* (n = 3)	0.02	86.99 ± 0.04	0.01	86.98 ± 0.03	0.02	87.00 ± 0.03	0.02	86.99 ± 0.04
*M. caprae* (n = 6)	0.02	86.57 ± 0.04	0.02	86.58 ± 0.05	0.01	86.62 ± 0.04	0.03	86.59 ± 0.06
*M. bovis* (n = 6)	0.02	86.58 ± 0.05	0.02	86.60 ± 0.05	0.02	86.64 ± 0.04	0.03	86.60 ± 0.07
*M. microti* (n = 7)	0.02	86.74 ± 0.07	0.02	86.73 ± 0.05	0.01	86.75 ± 0.05	0.03	86.74 ± 0.07

### HRM Analysis with Clinical Specimen**s**

**Figure 2:**
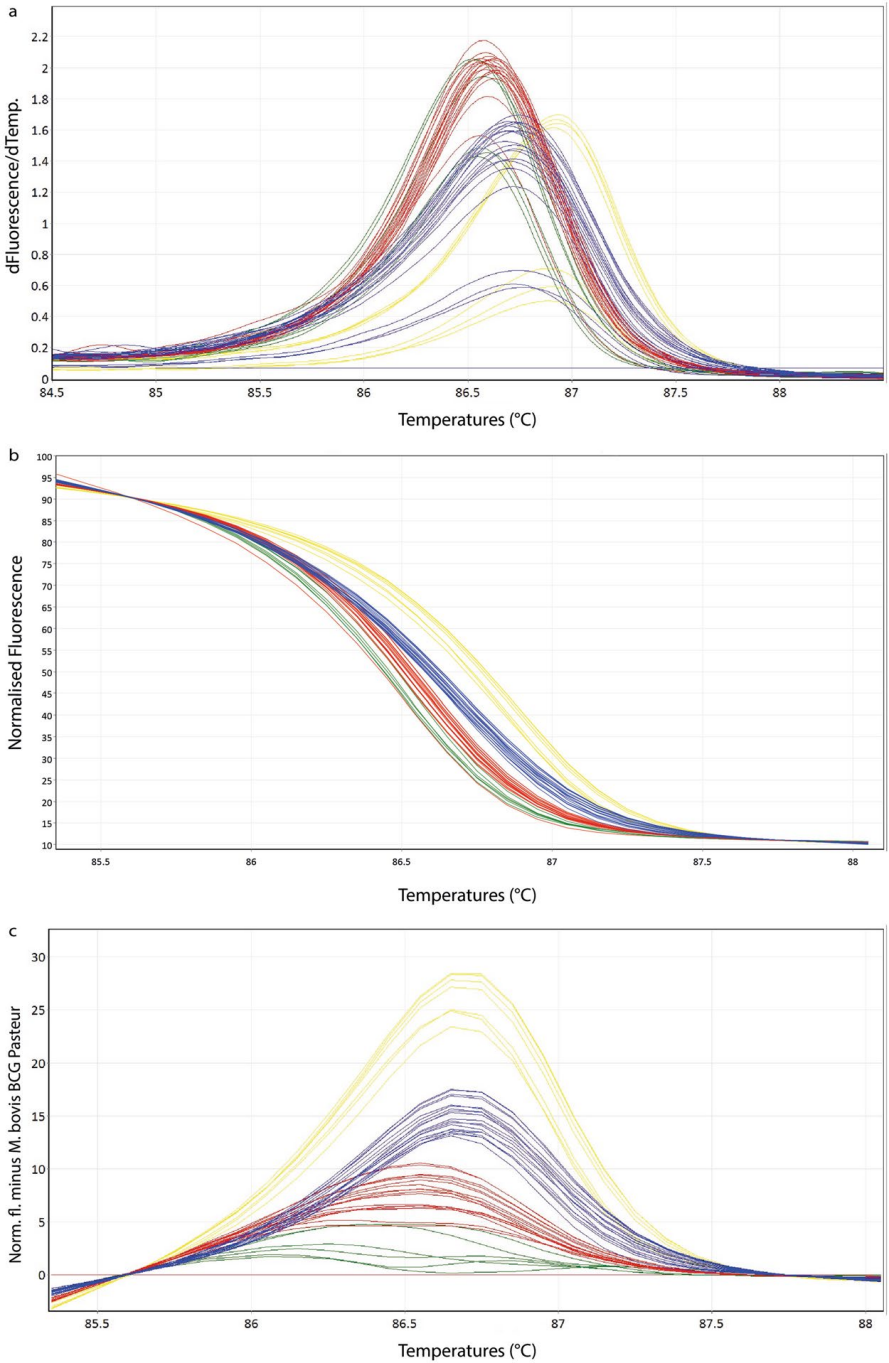
Representative high resolution melting graphs corresponding to one high resolution melting analysis of a subset of clinical specimens (n = 19). Curves of tested samples previously identified as *M. tuberculosis* are shown in yellow, *M. microti* in blue, *M. bovis/M. bovis* BCG in red and *M. caprae* in green. (**a**) Melting curves; (**b**) Normalized plot; (**c**) Difference plot.

25 clinical specimens were tested in the HRM assay and resulted in three main groups consistent with the expected MTBC species. The obtained normalized and difference plots of the tested subset of clinical specimens showed a clear discrimination (Fig. 2b,c). The intra-assay CVs (Table 2, Supplementary Table S2) were between 0.01–0.02% for *M. tuberculosis* and *M. caprae*, 0.01% for *M. bovis* and 0.05% for *M. microti*. The inter-assay CVs (Table 2, Supplementary Table S2) were higher than in the cultured samples with values between 0.12–0.15%. From those 25 clinical specimens 23 (92%) showed concordant identification results. Two samples revealed lower T_m_ values at approximately 84 °C, compared to the other sample results. The corresponding culture of one of those two clinical specimens (sample 17–2287) showed a correct species identification by HRM. The second clinical specimen (sample 17-1063) is not successfully cultured yet and further investigations are still ongoing.

**Table 2:** Melting temperatures (mean and standard deviation) of the intra- and inter-assay of a randomly chosen subset of clinical specimens for different MTBC species with its corresponding coefficients of variation (CV) in % are listed.

	Run 1		Run 2		Run 3		Inter-Assay	
	CV%	Tm values	CV%	Tm values	CV%	Tm values	CV%	Tm values
*M. tuberculosis* H37Rv		86.80		86.93		86.75		
*M. bovis* BCG Pasteur ATCC 35734		86.38		86.55		86.37		
*M. microti* ATCC 19422		86.52		86.70		86.48		
*M. tuberculosis* (n = 2)	0.01	86.73 ± 0.01	0.02	86.91 ± 0.03	0.02	86.70 ± 0.03	0.13	86.80 ± 0.13
*M. caprae* (n = 5)	0.01	86.27 ± 0.17	0.02	86.45 ± 0.15	0.02	86.34 ± 0.12	0.12	86.35 ± 0.25
*M. bovis* (n = 6)	0.01	86.41 ± 0.06	0.01	86.59 ± 0.04	0.01	86.36 ± 0.04	0.15	86.48 ± 0.16
*M. microti (*n = 7)	0.03	86.59 ± 0.04	0.02	86.73 ± 0.05	0.02	86.48 ± 0.05	0.14	86.60 ± 0.17

### Analytical Specificity

In the exclusivity run the tested 41 NTMs and *Nocardia paucivorans*, *Escherichia coli* and *Streptococcus suis* either showed no melting curves or melting curves with completely different T_m_ values compared to those obtained from samples of the MTBC. Therefore, the assay showed an analytical specificity of 100%.

### Limit of Detection

The limit of detection (LOD) for the lowest dilution of which the acceptance criteria were fulfilled was 10 genome equivalents (GE) for *M. tuberculosis, M. bovis/M. caprae* and *M. microti* (Table 3).

**Table 3:** Limit of detection of the real-time PCR step within the HRM assay.

MTBC Member	Genome equivalents	Ct	SD
*M. tuberculosis* H37Rv	1'000'000	16.42	0.10
	100'000	19.89	0.02
	10'000	23.76	0.07
	1000	27.48	0.08
	100	31.21	0.32
	10	35.04	0.27
	1	—	—
*M. bovis* BCG pasteur ATCC 35734	1'000'000	15.13	0.20
	100'000	18.61	0.16
	10'000	22.05	0.16
	1000	25.75	0.17
	100	29.38	0.20
	10	33.15	0.50
	1	35.51	0.12
*M. microti* ATCC 19422	1'000'000	15.73	0.06
	100'000	19.03	0.05
	10'000	22.98	0.07
	1000	26.28	0.13
	100	30.18	0.17
	10	33.92	0.14
	1	37.82	0.73

Determination of Ct values and its standard deviation (SD) of 3 replicates for a dilution series ranging from 1 to 1'000'000 genome equivalents using the three reference strains *M. tuberculosis* H37Rv, *M. bovis* Pasteur ATCC 35734 and *M. microti* ATCC 19422.

### Efficiency

The efficiencies of the RT-PCR were 87% for *M. microti*, 94% for *M. bovis* and 85% for H37Rv (Supplementary Fig. S1).

## Discussion

This study reports the development of a HRM assay to identify and distinguish the main members of the MTBC *c*omplex of clinical specimens and cultured samples in approximately two hours. *M. microti, M. tuberculosis/M. africanum* and *M. bovis/M. caprae* can be clearly and reliably distinguished from each other by unique difference plots. The T_m_ alone is not sufficient to discriminate the main species because of partially overlapping T_m_ values (Tables 1 and 2). However, after appropriate transformation of the melting curves into normalized and difference plots by applying algorithms of the Rotor-Gene Q Software 2.3.1 (Qiagen Hilden, Germany), the members of the MTBC can be clearly distinguished into three groups (Figs 1c and 2c). Using this strategy, the species-specific melting profiles showed an unambiguous picture.

To date, MTBC species identification is often based on methods requiring cultured samples^10^ or based on time-consuming procedures^11^. Halse *et al*. reported the development of a multiplex RT-PCR method for clinical specimens, however, this assay is more expensive due to the need of five different probes^16^. Furthermore, the complexity of such a multiplex reaction can be challenging when analysing tissue samples comprising various substances containing large amounts of co-extracted host DNA and ingredients, which can lead to inhibition of the PCR reaction^31^. Other studies evaluated mainly cultured isolates^13–15,17^. The main advantage of our developed HRM assay compared to previous studies is the implementation of a relatively cheap and straightforward singleplex method for directly extracted clinical specimens.

The current HRM assay identified MTBC positive cultured samples in complete agreement with results of the GenoType MTBC test (Hain Lifescience). The clinical specimens showed a concordance of 92%. The remaining 8% (n = 2) showed unspecific melting curves not allowing to assign the samples to MTBC using the developed method. Both samples derived from alpaca tissues, either from spleen or from a mix of different tissues including lymph node, lung, heart, liver and cervical vertebra. All 23 clinical samples lead to an unambiguous and correct result derived from lymph nodes, lung or liver tissues (Supplementary Table S3). It is likely that the content of these particular alpaca tissues interfered with the melting procedure. Further investigations to clarify this finding are continuing.

The intra- and inter-assay CVs showed very low values demonstrating a very good reproducibility of the method. The analytical specificity displayed a perfect value of 100% indicating a MTBC specific assay. Furthermore, the assay demonstrated a good PCR efficiency of more than 85% and a good sensitivity with a LOD of 10 GE.

One limitation of the assay is its inability to distinguish between *M. bovis, M. bovis* BCG and *M. caprae* with this particular primer set. Moreover, *M. africanum* cannot be separated from *M. tuberculosis*. In order to design a HRM assay having a high resolution detecting SNPs, the PCR amplicon should optimally not exceed 150 bp since longer amplicons would have a negative impact on the resolution of the assay. SNPs distantly located within a gene, as in the case of *gyrB*, are impossible to analyse by HRM using just one primer pair. Our primary goal was to clearly distinguish *M. microti* from other members of the MTBC complex since its proper identification in directly extracted clinical samples are advantageous considering its long cultivation time. Therefore, our developed HRM assay was restricted to the detection of 2 out of 5 possible SNPs within the *gyrB* gene^10^ resulting in a clear and rapid identification and differentiation of the three main MTBC species most relevant to veterinarians. In order to overcome the described limitation, there is the possibility to extend the HRM assay with a second primer pair, resulting in a two-reaction HRM paradigm, targeting a region to further discriminate *M. bovis* from *M. caprae* and *M. africanum* from *M. tuberculosis*. In addition, another drawback of the developed assay seems to lie in the failure of detection of samples deriving from certain alpacas (two out of four tested alpacas), especially samples containing spleen or bone.

## Conclusions

The developed HRM assay enables the simultaneous identification and differentiation of MTBC between the three clinically most relevant groups namely *M. tuberculosis/M. africanum*, *M. microti* and *M. bovis/M. caprae* from tissue samples as well as from cultured material. Therefore, the use of this powerful assay may save several months of cultivation time to differentiate between species of MTBC. It is an easy to perform, cheap, sensitive and specific assay leading to a result in less than two hours. Since tuberculosis is one of the top 10 causes of death worldwide, it is expected that a cost-effective and easy to set-up assay could be implemented in laboratories with moderate resources as a high-throughput screening and confirmatory tool for MTBC infections. This would significantly contribute to develop efficient public health and veterinary surveillance strategies worldwide.

## Methods

### Ethics statement

This study was carried out in accordance with the recommendations of Swiss federal regulations (TSV 916.401 and VSFK 817.190). Analysis of animal specimens was carried out within an official context of monitoring bovine tuberculosis and NTM infections, meaning that no animals were killed for the purposes of this research project and ethical approval was not necessary.

### Reference strains and samples

62 MTBC positive samples originating from 40 different animals and tissues (Table 4, Supplementary Table S3) were used for assay development. One additional isolate of a wild boar was kindly provided by Lucía de Juan Ferré and Beatriz Romero Martinez from the European Union Reference Laboratory for Bovine Tuberculosis in Madrid. Finally, a total of 63 samples comprising 38 isolates and 25 clinical specimens were tested. Reference strains *M. microti* ATCC 19422, *M. bovis* BCG Pasteur ATCC 35734 and *M. tuberculosis* H37Rv were included as positive controls in each run. To determine the specificity of the optimized HRM assay a set of 41 different non-tuberculous mycobacteria (NTM) was additionally tested (Supplementary Table S4). Moreover, *Nocardia paucivorans*, *Escherichia coli* and *Streptococcus suis* were included in this exclusivity panel in order to test for any non-specific signals.

**Table 4:** MTBC positive samples used for the development of the HRM method.

Species	Host	No. of isolates
**cultured material (n = 38)**		
*M. tuberculosis*	elephant	3
*M. caprae*	cow	7
*M. bovis*	cow	15
*M. microti*	cat	7
*M. microti*	alpaca	3
*M. microti*	llama	2
*M. microti*	wild boar (Spain)	1
**clinical specimens (n = 25)**		
*M. tuberculosis*	elephant	2
*M. caprae*	cow	5
*M. bovis*	cow	7
*M. microti*	cat	5
*M. microti*	alpaca	4
*M. microti*	llama	2
**Total**	63	

38 isolates obtained from cultured material, whereas 25 samples were clinical specimens directly extracted from tissue samples. 62 samples derived from Switzerland whereas one isolate originated from Spain.

### Culture and DNA extraction

Sample preparation, culture and DNA extraction were proceeded as described previously^6^. Briefly, genomic DNA was extracted harvesting mycobacteria from 1.5 ml of MGIT subcultures by centrifugation for 10 min at 13,000 × *g*. The sediment was suspended in 180 µl ATL buffer (Qiagen), transferred onto a bead beating matrix in a 2 ml microtube (Omni International, Kennesaw, USA), heat inactivated and subjected to mechanical cell lysis using a TissueLyser II (Qiagen) and enzymatic digestion with Proteinase K (Qiagen). Automated DNA preparation was performed on the QIAcube instrument using the QIAamp cador Pathogen Mini Kit protocol (Qiagen). DNA concentration was measured using a NanoDrop 2000c Spectrophotometer (Thermo Fisher Scientific, Reinach, Switzerland) and stored at −20 °C until use. DNA obtained from pure mycobacterial cultures were identified as MTBC using *artus M. tuberculosis* RG PCR Kit (Qiagen). Species identification of cultured samples was performed by GenoType MTBC test (Hain Lifescience). Clinical specimens were tested by Spoligotyping^32^ and multi-locus variable number tandem repeat analysis using an internationally established 24-loci panel^33^. Standard biosecurity procedures have been carried out for handling of samples. Cultures involving MTBC or NTM isolates were performed at the Biosafety Level 3 facility until heat deactivation. Sample preparation and DNA extraction were carried out under Biosafety Level 2 containment.

### HRM development and optimisation

**Figure 3:**
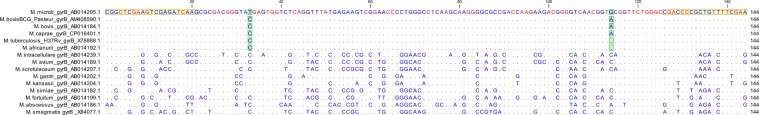
Sequence alignment of the amplicon within *gyrB* generated by the real-time PCR of the high resolution melting. Primer regions are indicated in yellow. Red letters and dots represent conserved bases whereas blue letters show areas with substitutions. The two single nucleotide polymorphisms distinguishing the main members of the *Mycobacterium tuberculosis* complex detected by the high resolution melting assay are highlighted with green.

A primer pair was designed specifically targeting a conserved region for MTBC on the *gyrB* gene. The forward HRM_gyrB_for (5′-CGGCTCGAAGTCGAGATCAAG-3′) and reverse HRM_gyrB_rev (5′-TTCGAAAACAGCGGGGTCG-3′) primers flank a 144 base pair (bp) amplicon. In contrast, other closely and distantly related NTM have a greater variability in the primer region as well as in the whole 144 base pair amplicon (Fig. 3).

The HRM assay was performed on the Rotor-Gene Q system (Qiagen) with the Type-it HRM PCR Kit (Qiagen). The reaction was performed in a total volume of 15 µl. 1 µl of sample DNA was added to a reaction mixture containing 7.5 µl 2X Type-it HRM Mastermix containing EvaGreen DNA-binding dye (Qiagen), 0.5 µM final concentration of each primer (Microsynth AG, Balgach, Switzerland) and ultrapure water. The PCR thermocycling conditions were as follows: initial denaturation at 95 °C for 5 min, 40 cycles with denaturation at 95 °C for 10 s and annealing/extension at 55 °C for 30 s followed by a second cycling step at 95 °C for 10 s and 40 °C for 2 min followed by a HRM ramping from 80 °C to 93 °C. Fluorescence data were acquired at 0.1 °C increments every 2 s to generate specific melting curves. For each experiment, the three reference strains *M. microti* ATCC 19422, *M. bovis* BCG Pasteur ATCC 35734 and *M. tuberculosis* H37Rv were included as melting curve standards and positive controls. To exclude contaminations in the reaction mixture, ultrapure water was added as a negative control in each experiment.

Data analysis was performed using Rotor-Gene Q Software 2.3.1 (Qiagen). Normalized and difference plots were generated. To normalize the results, the pre-melt (initial fluorescence) and post-melt (final fluorescence) signals of all samples were set to uniform relative values from 100% to 0%. In order to generate difference plots, normalized fluorescence data of sample curves were subtracted from a reference curve of *M. bovis* BCG Pasteur ATCC 35734 to visually accentuate differences in a greater resolution. The threshold value for peak calling was set at 0.5 dF/dT. In order to alleviate false negative results due to inhibition, clinical specimens were tested in duplicate undiluted and as a 1:5 dilution. The cultured samples were tested at concentrations between 100 pg and 10 ng.

To examine the intra- and inter-assay CV of the T_m_, representing the repeatability of the developed HRM method, a randomly chosen subset of 22 cultured and 19 clinical specimens were tested in triplicates in three independent runs at three different days.

### Analytical Specificity

In order to proof the analytical specificity of the primers an exclusivity panel including NTMs, *Nocardia paucivorans*, *Escherichia coli* and *Streptococcus suis* were tested.

### Efficiency and limit of detection

The efficiency and the analytical sensitivity of the RT-PCR were evaluated by triplicate testing of a 10-fold serial dilution series of each of the three reference strains. With an estimated genome size of 4.4 Mb, a DNA quantity of 4.8 fg was calculated for one GE of MTBC. The limit of detection was determined as lowest dilution with amplification of all triplicates with a standard deviation of ≤0.5.

## Supplementary information

Supplementary information

## Data Availability

Data generated during the study is presented in an analysed format in this manuscript. Raw datasets generated from the intra- and inter-assays are included in the Supplementary Information file. Additional raw data are available from the corresponding author on reasonable request.
