# Supplementary material for: Development of a new High Resolution Melting (HRM) assay for identification and differentiation of Mycobacterium tuberculosis complex samples
Source: Sci Rep. 2019 Feb 12;9:1850. doi: 10.1038/s41598-018-38243-6 (PMC6372708; doi:10.1038/s41598-018-38243-6)
Supplement: Supplementary file 1 — Supplementary information [file 41598_2018_38243_MOESM1_ESM.pdf]

Development of a new High Resolution Melting (HRM) assay for identification and differentiation of *Mycobacterium tuberculosis* complex samples

Patricia Landolt, Roger Stephan, Simone Scherrer

Supplementary information

**Supplementary Table [S1]. ** Raw data set and statistical parameters generated from the intra- and inter-assay using a randomly chosen subset of 22 cultured samples.

Isolate			Run 1						Run 2						Run 3						Inter-Assay			
MTBC member	sample no	origin	Ct	Tm	Mean Tm	SD	**CV%**	**Intra-assay CV%**	Ct	Tm	Mean Tm	SD	**CV%**	**Intra-assay CV%**	Ct	Tm	Mean Tm	SD	**CV%**	**Intra-assay CV%**	Mean Tm	SD	**CV%**	**Inter-assay CV%**
M. microti	ATCC 19422		23.08	86.75					23.15	86.72					23.38	86.73					86.73	0.02	**0.02**	
M. bovis BCG Pasteur	ATCC 35734		28.46	86.60					28.63	86.58					29.46	86.58					86.59	0.01	**0.01**	
M. tuberculosis	H37Rv		25.80	86.93					26.06	86.98					26.36	86.95					86.95	0.03	**0.03**	
M. tuberculosis	15-961 2B	elephant	18.47	86.97	86.96	0.01	**0.01**		18.50	87.00	87.00	0.00	**0.00**		18.89	86.98	86.98	0.02	**0.02**		86.98	0.02	**0.03**	
			18.49	86.95					18.41	87.00					18.97	86.97								
			18.47	86.95					18.53	87.00					18.95	87.00								
M. tuberculosis	15-1115-2	elephant	20.38	86.95	86.96	0.02	**0.02**		20.53	87.00	87.00	0.00	**0.00**		20.78	87.00	87.01	0.01	**0.01**		86.99	0.03	**0.03**	
			19.94	86.98					20.53	87.00					20.65	87.00								
			20.08	86.95					20.30	87.00					20.60	87.02								
M. tuberculosis	15-1221-1	elephant	23.48	86.97	86.99	0.03	**0.03**	**0.02**	23.74	86.98	86.97	0.02	**0.02**	**0.01**	23.98	86.98	87.00	0.02	**0.02**	**0.02**	86.99	0.02	**0.02**	**0.02**
			23.83	86.97					24.01	86.95					24.20	87.02								
			23.53	87.02					23.58	86.98					23.85	87.00								
M. caprae	14-13	cow	26.57	86.57	86.59	0.02	**0.02**		27.27	86.55	86.56	0.01	**0.01**		27.72	86.60	86.61	0.01	**0.01**		86.59	0.03	**0.03**	
			26.82	86.60					27.37	86.57					27.44	86.62								
			26.52	86.60					27.24	86.55					28.26	86.62								
M. caprae	22914	cow	23.99	86.58	86.58	0.00	**0.00**		24.29	86.57	86.55	0.02	**0.02**		24.46	86.65	86.64	0.01	**0.01**		86.59	0.04	**0.05**	
			23.87	86.58					24.23	86.53					24.75	86.63								
			23.97	86.58					24.33	86.55					24.41	86.63								
M. caprae	22848	cow	26.57	86.58	86.59	0.01	**0.01**		26.63	86.57	86.56	0.01	**0.01**		26.98	86.60	86.60	0.00	**0.00**		86.58	0.02	**0.03**	
			26.73	86.60					26.48	86.55					27.23	86.60								
			26.55	86.58					26.64	86.55					27.14	86.60								
M. caprae	22966	cow	23.75	86.55	86.58	0.03	**0.03**		23.66	86.60	86.58	0.02	**0.02**		24.22	86.58	86.59	0.01	**0.01**		86.58	0.00	**0.00**	
			23.00	86.60					23.61	86.58					24.03	86.60								
			23.44	86.60					23.75	86.57					23.91	86.58								
M. caprae	13-450	cow	21.29	86.58	86.58	0.01	**0.01**		21.39	86.57	86.60	0.03	**0.03**		21.48	86.58	86.60	0.02	**0.02**		86.59	0.01	**0.01**	
			21.23	86.57					21.35	86.60					21.47	86.60								
			21.29	86.58					21.31	86.62					21.57	86.62								
M. caprae	13-162	cow	25.11	86.58	86.56	0.03	**0.03**	**0.02**	25.41	86.63	86.63	0.01	**0.01**	**0.02**	25.82	86.58	86.60	0.02	**0.02**	**0.01**	86.60	0.03	**0.04**	**0.03**
			25.17	86.53					25.55	86.63					25.72	86.60								
			24.82	86.57					25.54	86.62					25.88	86.62								
M. bovis	20482	cow	26.55	86.55	86.56	0.01	**0.01**		26.61	86.62	86.61	0.02	**0.03**		26.86	86.62	86.63	0.02	**0.02**		86.60	0.03	**0.04**	
			26.39	86.57					26.64	86.62					26.96	86.65								
			26.31	86.57					26.28	86.58					26.81	86.62								
M. bovis	20594	cow	28.22	86.55	86.54	0.01	**0.01**		28.52	86.60	86.61	0.01	**0.01**		29.66	86.65	86.65	0.02	**0.02**		86.60	0.05	**0.06**	
			27.85	86.53					28.53	86.62					28.72	86.63								
			27.40	86.55					28.42	86.60					29.28	86.67								
M. bovis	20175	cow	22.06	86.55	86.58	0.03	**0.03**		21.80	86.58	86.57	0.02	**0.02**		22.20	86.62	86.64	0.02	**0.02**		86.60	0.04	**0.04**	
			21.30	86.58					21.72	86.58					22.22	86.65								
			22.02	86.60					21.95	86.55					22.43	86.65								
M. bovis	20606	cow	24.19	86.62	86.60	0.02	**0.02**		24.73	86.60	86.59	0.01	**0.01**		24.43	86.65	86.65	0.00	**0.00**		86.61	0.03	**0.04**	
			24.30	86.58					24.27	86.58					24.58	86.65								
			24.23	86.60					24.56	86.60					24.78	86.65								
M. bovis	22667	cow	22.92	86.60	86.61	0.01	**0.01**		22.91	86.60	86.62	0.03	**0.03**		23.33	86.65	86.64	0.01	**0.01**		86.62	0.02	**0.02**	
			22.87	86.62					23.20	86.65					23.46	86.65								
			23.06	86.60					23.03	86.60					23.43	86.63								
M. bovis	22539	cow	24.09	86.62	86.61	0.01	**0.01**	**0.02**	24.39	86.60	86.61	0.02	**0.02**	**0.02**	24.79	86.60	86.61	0.02	**0.02**	**0.02**	86.61	0.00	**0.00**	**0.03**
			23.91	86.60					24.59	86.60					24.67	86.63								
			24.31	86.62					24.24	86.63					25.08	86.60								
M. microti	22928	cat	20.62	86.70	86.73	0.03	**0.03**		20.57	86.75	86.76	0.01	**0.01**		20.79	86.73	86.73	0.03	**0.03**		86.74	0.02	**0.02**	
			20.63	86.75					20.76	86.77					20.53	86.70								
			20.72	86.73					20.86	86.75					20.74	86.75								
M. microti	15-1765	cat	24.74	86.72	86.72	0.02	**0.02**		24.84	86.78	86.76	0.02	**0.02**		25.31	86.75	86.75	0.00	**0.00**		86.74	0.02	**0.03**	
			24.48	86.73					24.74	86.75					25.42	86.75								
			24.96	86.70					24.76	86.75					25.15	86.75								
M. microti	14-58	cat	28.43	86.67	86.71	0.03	**0.04**		28.04	86.77	86.77	0.01	**0.01**		28.91	86.75	86.77	0.02	**0.02**		86.75	0.04	**0.04**	
			28.38	86.73					28.55	86.78					28.36	86.78								
			28.48	86.72					28.53	86.77					29.15	86.77								
M. microti	15-1955	cat	24.62	86.73	86.75	0.02	**0.02**		24.81	86.77	86.76	0.01	**0.01**		25.08	86.80	86.78	0.02	**0.02**		86.76	0.02	**0.02**	
			24.08	86.77					24.45	86.77					24.69	86.77								
			23.90	86.75					24.62	86.75					24.87	86.77								
M. microti	16-2156	cat	26.87	86.75	86.77	0.03	**0.03**		27.19	86.70	86.71	0.02	**0.02**		27.80	86.75	86.75	0.00	**0.00**		86.74	0.03	**0.04**	
			27.08	86.77					27.08	86.70					27.42	86.75								
			26.65	86.80					27.16	86.73					27.39	86.75								
M. microti	1522744	cat	26.98	86.77	86.77	0.01	**0.01**		27.26	86.73	86.70	0.03	**0.03**		27.78	86.77	86.76	0.01	**0.01**		86.74	0.04	**0.04**	
			26.92	86.77					26.96	86.70					27.39	86.75								
			27.21	86.78					27.18	86.68					27.55	86.75								
M. microti	16-1347	cat	23.04	86.75	86.78	0.03	**0.03**	**0.02**	23.05	86.72	86.73	0.02	**0.02**	**0.02**	23.49	86.75	86.73	0.02	**0.02**	**0.01**	86.75	0.03	**0.03**	**0.03**
			22.50	86.80					23.40	86.73					23.50	86.73								
			23.15	86.78					22.94	86.75					23.18	86.72								

Supplementary Table S2. Raw data set and statistical parameters generated from the intra- and inter-assay using a randomly chosen subset of 19 clinical specimens.

Clinical specimen			Run 1						Run 2						Run 3						Inter-assay			
MTBC member	sample no	origin	Ct	Tm	Mean Tm	SD	**CV%**	**Intra-assay CV%**	Ct	Tm	Mean Tm	SD	**CV%**	**Intra-assay CV%**	Ct	Tm	Mean Tm	SD	**CV%**	**Intra-assay CV%**	Mean Tm	SD	**CV%**	**Inter-assay CV%**
M. microti	ATCC 19422		23.07	86.52					22.75	86.70					22.68	86.48								
M. bovis BCG Pasteur	ATCC 35734		28.16	86.38					28.46	86.55					27.81	86.37								
M. tuberculosis	H37Rv		25.92	86.80					25.46	86.93					25.38	86.75								
M. tuberculosis	15-961 2	elephant	35.67	86.73	86.72	0.01	**0.01**		36.39	86.90	86.89	0.01	**0.01**		37.77	86.73	86.70	0.03	0.03		86.77	0.10	0.12	
			35.67	86.72					36.37	86.88					35.58	86.70								
			36.01	86.72					35.85	86.88					35.05	86.68								
M. tuberculosis	15-961 1	elephant	28.01	86.73	86.73	0.01	**0.01**	**0.01**	28.75	86.90	86.92	0.02	**0.02**	**0.02**	27.97	86.68	86.68	0.01	0.01	**0.02**	86.77	0.13	0.15	**0.13**
			28.57	86.72					28.63	86.93					28.10	86.68								
			28.47	86.73					28.32	86.92					28.29	86.67								
M. caprae	14-13	cow	>40.00	86.25	86.24	0.01	**0.01**		>40.00	86.45	86.46	0.01	**0.01**		>40.00	86.45	86.41	0.04	0.04		86.37	0.11	0.13	
			>40.00	86.23					>40.00	86.45					>40.00	86.38								
			>40.00	86.25					>40.00	86.47					>40.00	86.40								
M. caprae	22914	cow	33.14	86.43	86.41	0.02	**0.02**		33.14	86.60	86.57	0.03	**0.03**		32.18	86.35	86.34	0.02	0.02		86.44	0.12	0.13	
			33.15	86.40					33.00	86.55					33.34	86.35								
			33.49	86.40					33.64	86.55					32.35	86.32								
M. caprae	22848	cow	21.81	86.38	86.39	0.01	**0.01**		21.55	86.57	86.55	0.03	**0.03**		21.23	86.35	86.34	0.01	0.01		86.43	0.11	0.12	
			21.9	86.40					21.52	86.52					21.36	86.35								
			21.89	86.38					21.78	86.55					21.59	86.33								
M. caprae	13-162	cow	>40.00	86.28	86.28	0.00	**0.00**		38.38	86.48	86.46	0.02	**0.02**		>40.00	86.37	86.38	0.02	0.02		86.37	0.09	0.10	
			38.43	86.28					38.53	86.45					39.77	86.40								
			39.58	86.28					39.61	86.45					>40.00	86.38								
M. caprae	22971	cow	39.45	86.12	86.11	0.01	**0.01**	**0.01**	>40.00	86.30	86.32	0.03	**0.03**	**0.02**	>40.00	86.22	86.24	0.02	0.02	**0.02**	86.23	0.11	0.12	**0.12**
			>40.00	86.10					>40.00	86.35					>40.00	86.25								
			>40.00	86.12					>40.00	86.32					>40.00	86.25								
M. bovis	20593	cow	18.23	86.35	86.35	0.00	**0.00**		18.13	86.55	86.56	0.02	**0.02**		17.64	86.35	86.34	0.02	0.02		86.42	0.12	0.14	
			17.93	86.35					17.69	86.55					17.53	86.35								
			17.66	86.35					17.93	86.58					17.14	86.32								
M. bovis	20594	cow	15.47	86.40	86.40	0.00	**0.00**		15.30	86.60	86.59	0.02	**0.02**		15.39	86.35	86.34	0.01	0.01		86.44	0.13	0.15	
			15.44	86.40					15.27	86.60					15.31	86.33								
			15.3	86.40					14.99	86.57					14.98	86.35								
M. bovis	20609	cow	24.13	86.40	86.42	0.02	**0.02**		24.94	86.60	86.62	0.02	**0.02**		23.83	86.38	86.37	0.02	0.02		86.47	0.13	0.15	
			24.72	86.43					24.70	86.62					24.49	86.35								
			24.69	86.42					24.27	86.63					24.15	86.37								
M. bovis	20606	cow	15.44	86.45	86.46	0.01	**0.01**		15.19	86.62	86.63	0.01	**0.01**		14.85	86.40	86.39	0.02	0.02		86.49	0.12	0.14	
			15.12	86.47					14.96	86.63					14.63	86.37								
			15.64	86.47					15.40	86.63					14.90	86.40								
M. bovis	20596	cow	15.76	86.45	86.44	0.01	**0.01**		15.53	86.62	86.62	0.00	**0.00**		15.27	86.35	86.36	0.01	0.01		86.47	0.13	0.16	
			15.43	86.45					15.53	86.62					15.16	86.37								
			15.74	86.43					15.51	86.62					15.10	86.35								
M. bovis	20600	cow	15.84	86.47	86.46	0.02	**0.03**	**0.01**	15.38	86.60	86.58	0.02	**0.02**	**0.01**	14.96	86.33	86.33	0.00	0.00	**0.01**	86.46	0.13	0.15	**0.15**
			15.63	86.47					15.49	86.58					15.43	86.33								
			15.63	86.43					15.66	86.57					15.08	86.33								
M. microti	22928	cat	24.51	86.60	86.57	0.03	**0.03**		24.33	86.72	86.70	0.02	**0.02**		23.78	86.43	86.45	0.03	0.03		86.58	0.12	0.14	
			24.29	86.57					24.37	86.70					24.19	86.48								
			24.52	86.55					24.46	86.68					24.02	86.45								
M. microti	15-342	alpaca	23.14	86.55	86.57	0.03	**0.03**		22.84	86.70	86.71	0.04	**0.04**		22.67	86.45	86.48	0.03	0.03		86.59	0.12	0.14	
			23.17	86.57					22.94	86.68					22.64	86.48								
			23.04	86.60					22.56	86.75					22.69	86.50								
M. microti	17-1084	cat	26.85	86.57	86.56	0.01	**0.01**		27.01	86.68	86.70	0.03	**0.03**		26.48	86.50	86.51	0.01	0.01		86.59	0.10	0.12	
			26.64	86.55					26.85	86.70					26.64	86.50								
			26.92	86.57					26.74	86.73					26.59	86.52								
M. microti	15-1955	cat	29.81	86.55	86.58	0.03	**0.03**		29.44	86.70	86.71	0.01	**0.01**		28.95	86.48	86.47	0.02	0.02		86.59	0.12	0.14	
			29.55	86.58					29.63	86.72					28.99	86.45								
			29.13	86.60					29.65	86.72					29.16	86.48								
M. microti	14-690	alpaca	22.76	86.60	86.58	0.03	**0.03**		22.86	86.75	86.74	0.01	**0.01**		22.82	86.50	86.52	0.02	0.02		86.61	0.12	0.14	
			23.18	86.58					22.95	86.75					22.57	86.52								
			23.02	86.55					22.96	86.73					22.50	86.53								
M. microti	17-549	llama	32.87	86.58	86.60	0.02	**0.02**	**0.03**	33.75	86.77	86.75	0.03	**0.03**	**0.02**	33.28	86.52	86.49	0.03	0.03	**0.02**	86.61	0.13	0.15	**0.14**
			33.28	86.62					33.44	86.75					32.55	86.47								
			33.62	86.60					33.52	86.72					32.48	86.48								

Supplementary Figure S1. Real-time-PCR amplification curves and standard curves of the serial dilution using (a) *M. tuberculosis* H37Rv; (b) *M. bovis* BCG Pasteur ATCC 35734 and (c) *M. microti* ATCC 19422.

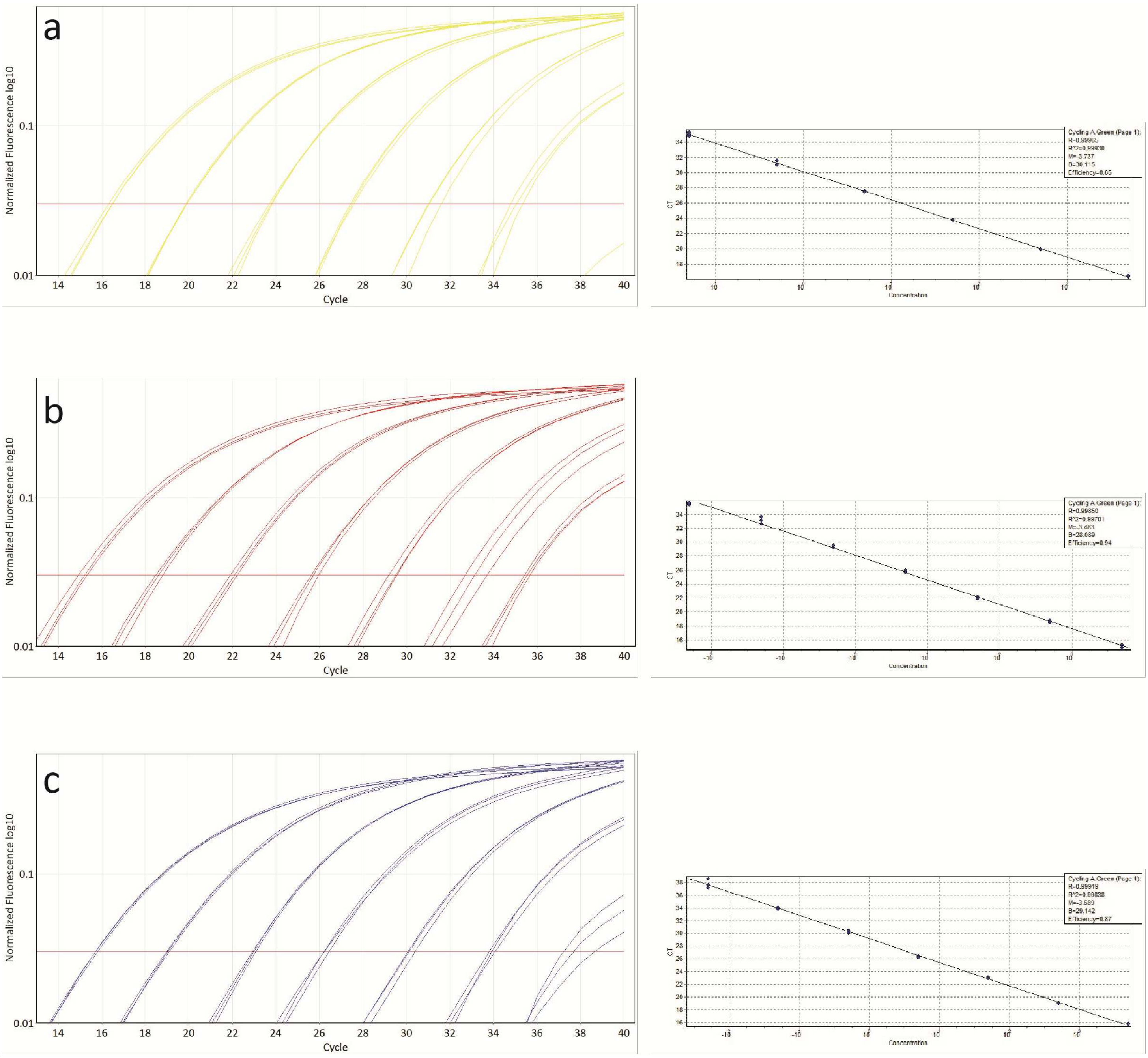

Supplementary Table [S3]**.** MTBC positive isolates used for the development of the high resolution melting assay.

Sample	Species	Origin	Specimen	Culture?	Clinical specimen?
M. bovis **(15)**					
20175	*M. bovis*	cow	lung	cultured	not available
20482	*M. bovis*	cow	lung, lymph node pool	cultured	not available
20531	*M. bovis*	cow	lymph node pool	cultured	not available
20593	*M. bovis*	cow	lymph node pool	cultured	clinical specimen
20594	*M. bovis*	cow	lymph node pool	cultured	clinical specimen
20596	*M. bovis*	cow	lymph node pool	cultured	clinical specimen
20597	*M. bovis*	cow	lymph node pool	cultured	not available
20599	*M. bovis*	cow	lymph node pool	cultured	not available
20600	*M. bovis*	cow	lymph node pool	cultured	clinical specimen
20606	*M. bovis*	cow	lymph node pool	cultured	clinical specimen
20608	*M. bovis*	cow	lymph node pool	cultured	clinical specimen
20609	*M. bovis*	cow	lymph node pool	cultured	clinical specimen
20665	*M. bovis*	cow	lymph node pool	cultured	not available
22539	*M. bovis*	cow	lymph node	cultured	not available
22667	*M. bovis*	cow	lymph node	cultured	not available
M. caprae **(8)**					
14-13	*M. caprae*	Cow	lymph node	cultured	clinical specimen
22914	*M. caprae*	Cow	lymph node pool	cultured	clinical specimen
22971	*M. caprae*	Cow	lymph node pool	cultured	clinical specimen
22848	*M. caprae*	Cow	liver, lung, lymph node pool	cultured	clinical specimen
22966	*M. caprae*	Cow	lymph node pool	cultured	not available
13-450	*M. caprae*	Cow	lymph node	cultured	not available
13-162	*M. caprae*	Cow	lymph node	cultured	not available
20948	*M. caprae*	Cow	lymph node	not available	clinical specimen
M. microti **(15)**					
22928	*M. microti*	cat	lung, lymph node pool	cultured	clinical specimen
15-1765	*M. microti*	cat	lung	cultured	clinical specimen
15-342	*M. microti*	alpaca	lymph node	cultured	clinical specimen
14-58	*M. microti*	cat	lung	cultured	clinical specimen
14-690	*M. microti*	alpaca	liver	cultured	clinical specimen
17-2287	*M. microti*	alpaca	spleen	cultured	clinical specimen
15817	*M. microti*	lama	lymph node	cultured	clinical specimen
15-1955	*M. microti*	cat	lymph node	cultured	clinical specimen
16-2156	*M. microti*	cat	bronchoalveolar lavage	cultured	not available
1522744	*M. microti*	cat	lung, lymph node pool	cultured	not available
16-1347	*M. microti*	cat	lung	cultured	not available
17-1084	*M. microti*	cat	lymph node, skin	culture ongoing	clinical specimen
17-549	*M. microti*	lama	liver	cultured	clinical specimen
17-1063	*M. microti*	alpaca	lymph node, lung, heart, liver, cervical vertebra	culture ongoing	clinical specimen
MI16	*M. microti*	wildboar (Spain)	unknown	cultured	not available
M. tuberculosis **(4)**					
15-961-2	*M. tuberculosis*	elephant 1	lung	cultured	clinical specimen
15-961-1	*M. tuberculosis*	elephant 1	pharyngeal swab	not cultivated	clinical specimen
15-1115-2	*M. tuberculosis*	elephant 2	lung	cultured	not available
15-1221-1	*M. tuberculosis*	elephant 3	lung	cultured	not available

Supplementary Table S4**.** An exclusivity panel consisting of 41 non-tuberculous mycobacteria and 3 non-mycobacterial species were tested for specificity of the high resolution melting assay.

Species	No. of isolates	Species	No. of isolates
M. abscessus sp.	2	*M. malmoense*	1
M. avium subsp. *avium*	2	*M. marinum*	1
M. avium subsp. *hominissuis*	32	*M. monacense*	3
M. avium subsp. *paratuberculosis*	1	*M. nebraskense*	1
M. avium subsp. *silvaticum*	1	*M. neoaurum*	5
M. bourgelatii	1	*M. nonchromogenicum*	7
M. celatum	1	*M. palustre*	1
M. chelonae subsp. *chelonae*	1	*M. parafortuitum*	2
M. chimaera/intracellulare/youngonense	1	*M. paragordonae*	6
M. chitae	1	*M. peregrinum*	2
M. elephantis	1	*M. persicum*	2
M. engbaekii	1	*M. phlei*	3
M. europaeum	1	*M. scrofulaceum*	1
M. fortuitum/porcinum	1	*M. simiae*	1
M. goodii	1	*M. smegmatis*	1
M. gordonae	2	*M. szulgai*	1
M. hassiacum	1	*M. terrae*	1
M. interjectum/paraense	1	*M. vaccae*	4
M. intermedium	1	*M. xenopi*	5
M. intracellulare	3	*Nocardia paucivorans*	1
M. kansasii	10	*Escherichia coli*	1
M. lymphaticum	1	*Streptococcus suis*	1
